# Multiple Swellings Over the Scrotum: Epidermal Inclusion Cysts

**DOI:** 10.7759/cureus.55830

**Published:** 2024-03-08

**Authors:** Bhushan Shah, Devendra S Yadav, Adithya Reddy, Jayant Bajaj

**Affiliations:** 1 General Surgery, Dr. D. Y. Patil Medical College, Hospital and Research Centre, Pune, IND; 2 General Surgery, Dr. D. Y. Patil Medical College, Hospital and Research Centre, Dr. D. Y. Patil Vidyapeeth (Deemed to be University), Pune, IND

**Keywords:** painless swelling, hemi scrotum, dermato-surgery, general plastic surgery, epidermal inclusion cyst

## Abstract

Epidermal inclusion cysts are lesions that are benign and commonly occur on the regions of the scalp, face, neck, and scrotum. It is usually a painless condition but may become painful if it gets infected.

A rupture of the cyst wall can lead to an intensely painful inflammatory reaction, and it is a common presentation to a surgeon.

In this case, the patient reported multiple painless swellings on the scrotum, which were excised under spinal anesthesia. It was initially thought to be trichilemmal cysts, but on histopathological examination (HPE), it was diagnosed as epidermal inclusion cysts.

## Introduction

Epidermal cysts, epidermal inclusion cysts, or follicular infundibular cysts are benign, rare, and occur commonly on the hair-bearing regions such as the scalp, face, neck, back, and scrotum [1-6]. On histopathological examination (HPE), these cysts show a stratified squamous epithelium lining with the presence of deposition of keratin, cholesterol crystals, and debris [1-6].

Usually, if the cysts are small and do not hamper any daily activities, they are left as they are and can be managed conservatively in cases of mild discomfort. Single cysts on the scrotum are relatively common, but multiple cysts are rare. It is even more uncommon to have multiple cysts that almost cover the entire scrotal skin. But if the cysts become painful and inflamed, they may rupture, causing pus discharge and infection of the scrotal wall [7]. So in this case, we were able to surgically excise the cysts before they could lead to any further complications.

## Case presentation

A male in his 70s, a farm worker by occupation, presented to the outpatient department (OPD) with complaints of the presence of multiple swellings over the scrotal region for seven months. Initially, the patient noticed two small swellings, which he overlooked and did not take any treatment for. After a period of two months, these swellings progressed over time to multiple large swellings. The swellings were not associated with pain.

**Figure 1:**
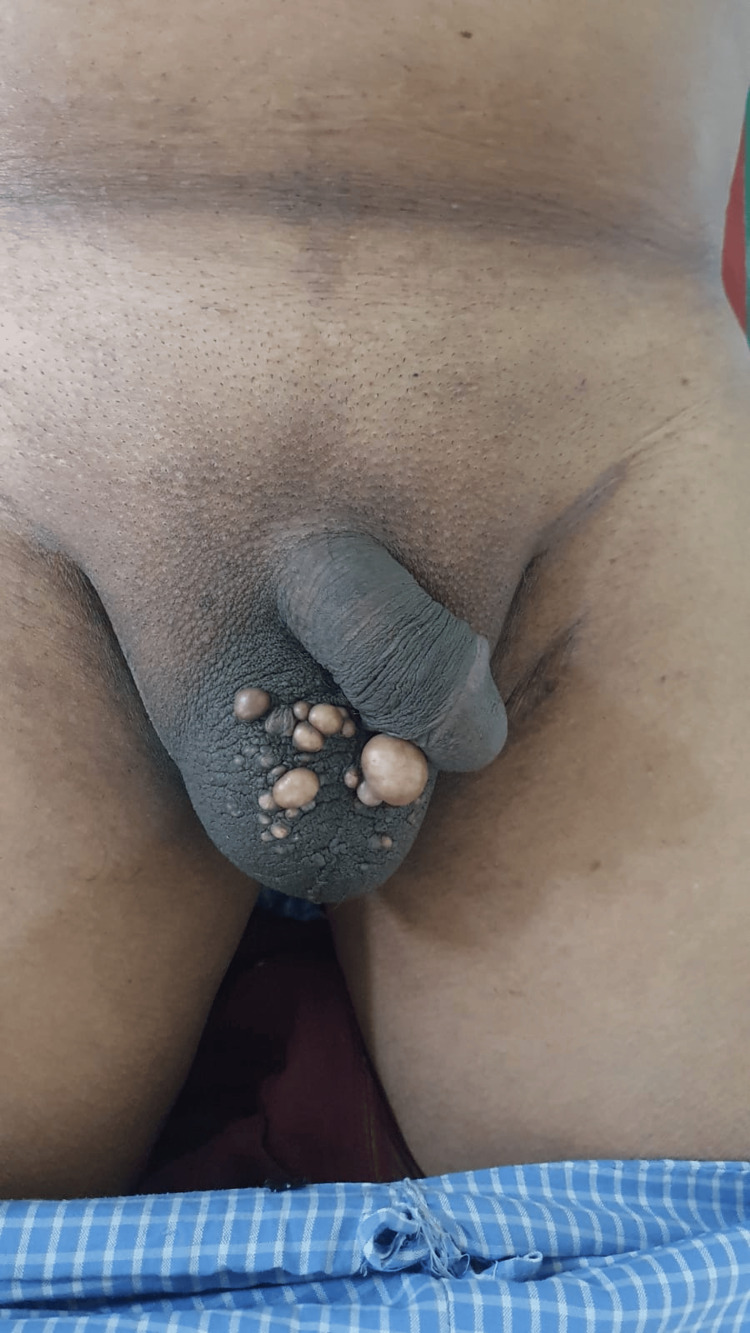
Image showing multiple sebaceous cysts over the scrotum.

On examination, multiple swellings of sizes 0.5 x 0.5 to 1.5 x 1.5 were present over the scrotum. The swellings were soft in consistency, with no local rise in temperature. There was no discharge, and no punctum was seen (Figure 1). The patient was posted for wide local excision of the scrotum under spinal anesthesia.

The patient had no intraoperative complications, and the surgery was uneventful. The patient was started on injection ceftriaxone postoperatively for five days, and the excised scrotal skin was sent for histopathological examination. Three tissue pieces were sent for histopathological examination.

**Figure 2:**
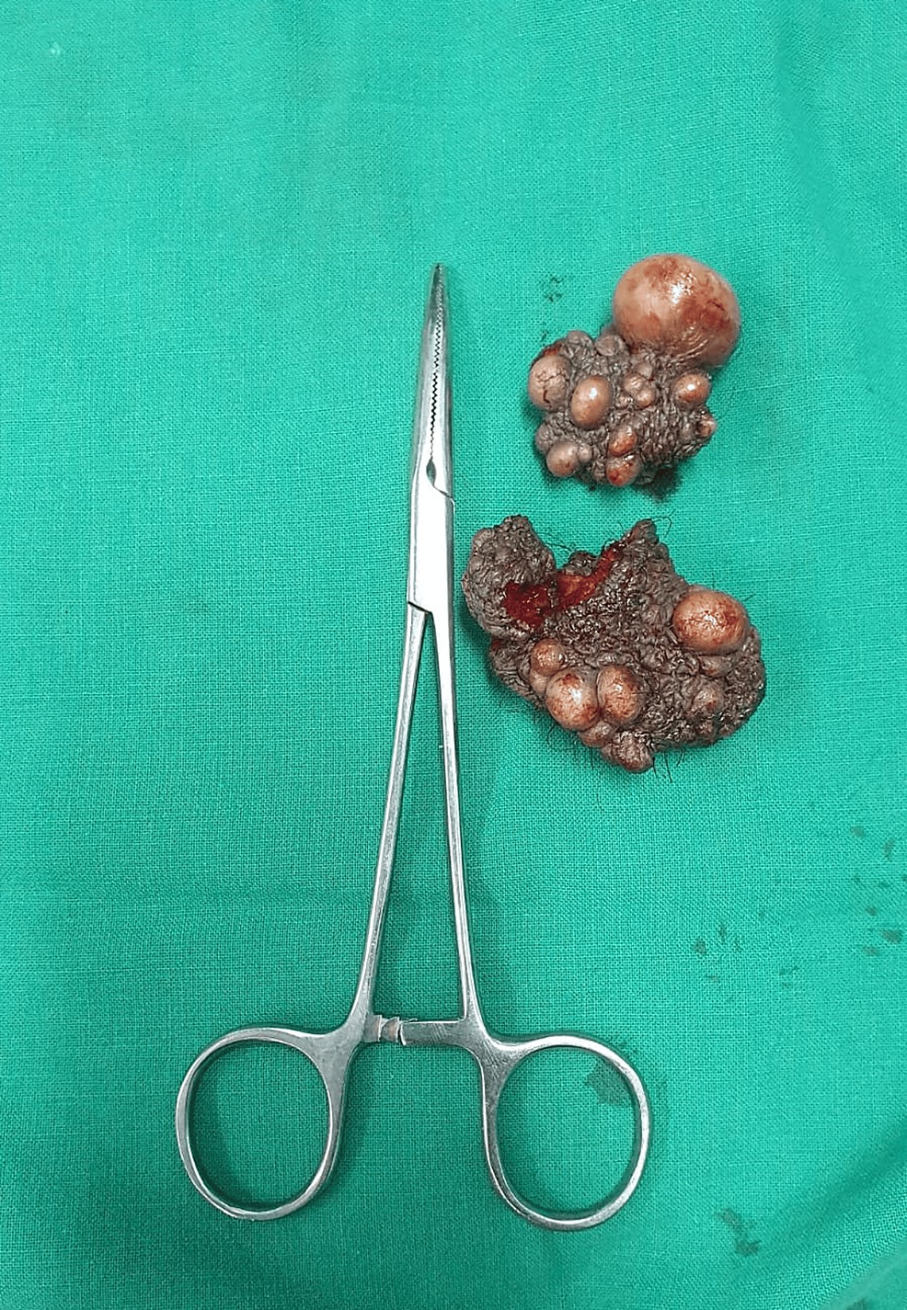
Image showing the 1st and 2nd tissue pieces sent for HPE. HPE: histopathological examination

The first piece measures 2.5 x 1.5 x 0.5 cm (the smallest piece). The skin flap is noted to measure 2.5 x 1.5 x 0.5 cm, along with the hair follicles (Figure 2). The second piece measures 3.5 x 2.5 x 1.8 cm. Externally, the skin flap was noted to measure 2.5 x 2.5 cm, along with the hair follicles. Multiple cyst-like structures are seen (globular in appearance) ranging from 0.4 to 2.5 cm in diameter (Figure 2).

**Figure 3:**
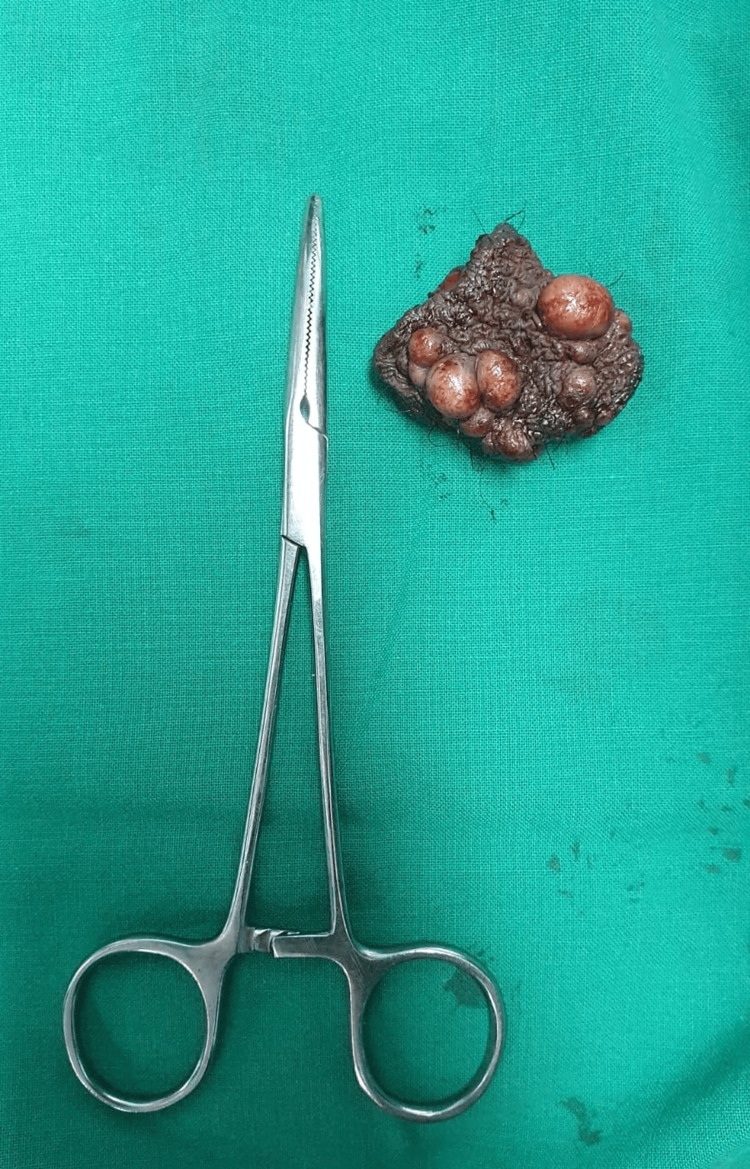
Image showing the 3rd (largest) tissue piece sent for HPE. HPE: histopathological examination

The third piece measures 6 x 3.6 x 1.5 cm (the largest piece). The skin flap is noted to measure 6 x 3.6 cm, along with the hair follicles. Multiple cyst-like globular swellings were noted, ranging from 0.5 to 1.2 cm (Figure 3).

**Figure 4:**
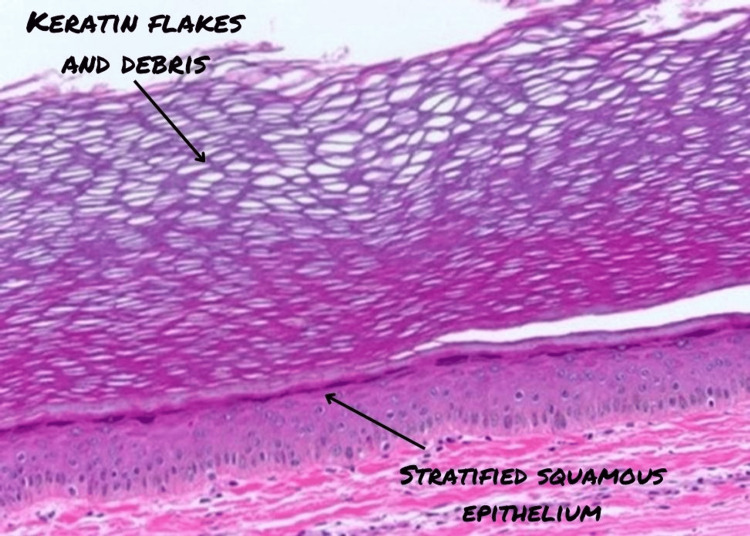
Microscopic image showing keratin formation.

**Figure 5:**
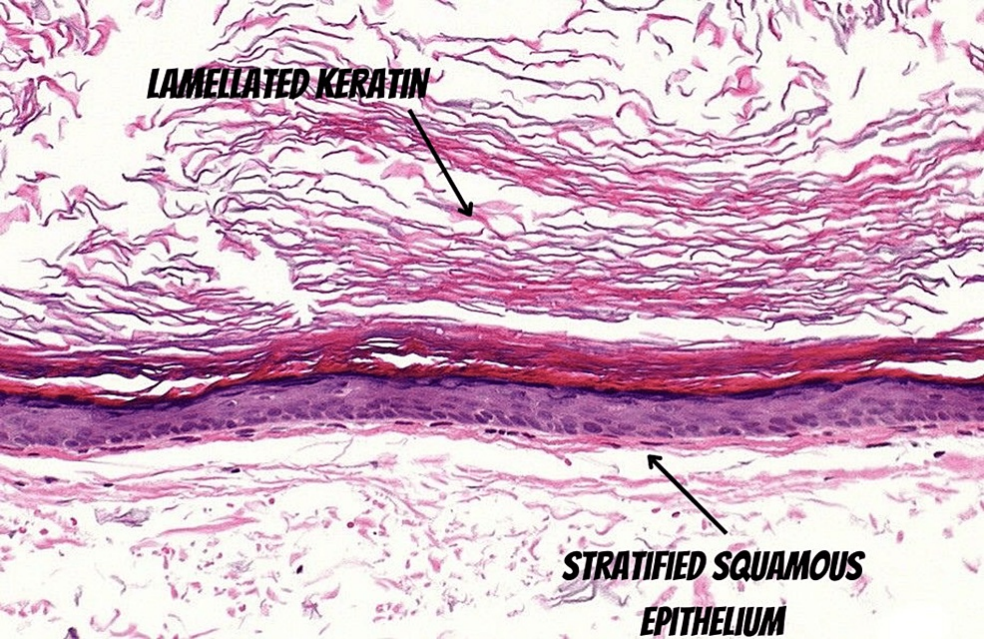
Microscopic image showing lamellated keratin.

On microscopy, multiple random sections studied showed skin tissue lined by stratified squamous epithelium with multiple invaginations containing keratinous flakes and debris (Figure 4). The cyst also showed areas of lamellated keratin. (Figure 5).

The histopathological diagnosis was suggestive of multiple epidermal inclusion cysts.

The surgery was uneventful, and the patient was discharged after seven days. The patient’s suture line was healthy, and the patient was followed up regularly for dressing. The patient had no further complaints, and he was able to return to his day-to-day activities without any complications.

## Discussion

Epidermoid cysts (often mistakenly referred to as sebaceous cysts) are classically the result of keratin-plugged pilosebaceous units. They commonly affect adult men and women and present as a dermal or subcutaneous cyst with a single, keratin-plugged punctum at the skin surface, often at or above the upper chest and back. Epidermoid cysts are the most common cutaneous cysts and are histologically characterized by a mature epidermis complete with a granular layer [8].

Epidermal inclusion cysts are benign lesions that show a stratified squamous epithelium lining in the presence of deposition of keratin, cholesterol crystals, and debris. The cysts are usually asymptomatic unless they get large and interfere with the function, get infected, or rupture. An infected cyst can become painful and may even rupture, leading to pus discharge. If a single cyst is infected, it can be drained without any complications. However, if left untreated, the infection can spread to other cysts nearby and eventually reach the scrotal wall, which may lead to Fournier's gangrene and sepsis [8]. Malignant transformation of these cysts is usually rare.

The differential diagnosis of these cysts usually includes scrotal calcinosis and trichilemmal cysts, which are differentiated based on histopathological examination.

Ultrasonography (USG) is the usual investigation done for epidermal inclusion cysts. On USG, the lesions look well-circumscribed, round, or oval hypoechoic lesions with no evidence of internal blood flow on the Doppler [9]. Along with ultrasonography, MRI is another investigation that is done for scrotal mass. On MRI, epidermal inclusion cysts are seen as well-defined solid masses with a low-signal capsule.

## Conclusions

Epidermal inclusion cysts are benign lesions with low malignant potential that can be easily diagnosed with USG or MRI due to their roundish shape and avascular center. Although not to be confused with other swellings of the scrotum, histopathological examination helps to differentiate epidermal inclusion cysts from trichilemmal cysts and scrotal calcinosis.

These cysts can be surgically excised using minimal excision techniques as the healing time is shorter and there are no to minimal complications, which is ideal for cysts over the scrotum. So early intervention can provide symptomatic relief to the patient and prevent potential complications that might prove fatal later.
